# Elevated fecal and serum calprotectin in COVID-19 are not consistent with gastrointestinal symptoms

**DOI:** 10.1038/s41598-021-01231-4

**Published:** 2021-11-09

**Authors:** Hajar Shokri-Afra, Ahmad Alikhani, Bahman Moradipoodeh, Farshid Noorbakhsh, Hafez Fakheri, Hemen Moradi-Sardareh

**Affiliations:** 1 Gut and Liver Research Center, Non-Communicable Diseases Institute, Mazandaran University of Medical Sciences, Sari, Iran; 2 Department of Infectious Diseases, Antimicrobial Resistance Research Center and Communicable Diseases Institute, Mazandaran University of Medical Sciences, Sari, Iran; 3 Department of Laboratory, Islamic Azad University, Lahijan Branch, Lahijan, Iran; 4 Department of Immunology, Tehran University of Medical Sciences, Tehran, Iran; 5 Asadabad School of Medical Science, Asadabad, Iran; 6 BioMad As Company, Oslo, Norway

**Keywords:** Biochemistry, Immunology, Biomarkers, Health care, Medical research

## Abstract

Intestinal epithelial cell damage caused by SARS-CoV-2 infection was thought to be associated with gastrointestinal symptoms and decreased fecal consistency. The association of the gastrointestinal symptoms with the COVID-19-mediated inflammatory response triggered by the gastrointestinal immune system was investigated in this paper. Intestinal inflammation marker fecal calprotectin along with serum calprotectin and other inflammatory markers were measured in COVID-19 cases with and without GI manifestations as well as healthy individuals. Analyses were performed to compare COVID-19 patient subgroups and healthy controls and examine the relationship between fecal and serum calprotectin levels with gastrointestinal symptoms and disease severity. COVID-19 patients (*n* = 70) were found to have markedly elevated median levels of fecal (124.3 vs. 25.0 µg/g; *P* < 0/0001) and serum calprotectin (3500 vs. 1060 ng/mL; *P* < 0/0001) compared with uninfected controls. Fecal and serum calprotectin levels were not significantly different between COVID-19 patients who displayed GI symptoms and those who did not. Compared with other acute phase markers, both fecal and serum calprotectin were superior in identifying COVID-19 patients who progressed to severe illness. Although the progression of COVID-19 disease is marked by an elevation of fecal and serum calprotectin, gastrointestinal symptoms or diarrhea were not correlated with calprotectin increase level.

## Introduction

Coronavirus disease 2019 (COVID-19) is caused by severe acute respiratory syndrome coronavirus 2 (SARS-CoV-2)^1^. The clinical findings of COVID-19 vary widely, from nothing to a life-threatening illness. Common symptoms include fever, muscle pain, weakness, cough, acute respiratory distress syndrome (ARDS), and respiratory failure^2–6^. Even though gastrointestinal (GI) symptoms are observed in up to 60% of COVID-19 patients, their pathophysiology is not well understood^7,8^.

A growing body of evidence points to a dysregulated immune response that leads to cytokine storm characterized by elevated circulating cytokines and participates in the sudden deterioration of COVID-19 patients. In addition, tissue damaged by SARS-CoV-2 infection induces the recruitment and activation of inflammatory cytokine-producing immune cells, especially neutrophils and macrophages, in an amplifying loop^9^. A marked neutrophilia is associated with COVID-19 severity which in turn, induces a massive release of S100A8/S100A9 calprotectin^10^.

Recent meta-analysis with eight included studies as well as other study reported that serum calprotectin (SC) was increased in COVID-19 patients, especially in cases admitted to the intensive care units (ICU)^11,12^. Previously, calprotectin was introduced as a diagnostic biomarker for intestinal mucosal damage^13^. Numerous studies have suggested that neutrophils migration into the GI tract due to inflammatory processes is the cause of elevated fecal calprotectin (FC)^2,14^. It has been recently proposed that SARS-CoV-2 infects GI tract cells and subsequently cause continuous production of cytokines such as interleukin 6 (IL-6)^15^. Accordingly, large production of FC may be expected following GI infection by SARS-CoV-2^16^.

Few studies have dealt with FC in COVID-19 patients, moreover, conflicting results have been mentioned^16^. However, no research has yet investigated the association between serum and faecal levels of S100A8/S100A9 in COVID-19. We aimed to evaluate SC and FC levels simultaneously in COVID-19 patients with and without GI symptoms. Here, we also showed the relation of SC and FC with disease diagnosis and severity prognosis.

## Results

### Patient demographics and clinical characteristics

In the present study of the 89 individuals, 70 were confirmed COVID-19 cases and 19 individuals were healthy. As shown in Table 1, there were no significant differences in gender between COVID-19 patients and the healthy group (*P* = 0.363). Patients in the COVID-19 group had an average age of 51.47 ± 17.28 years, and cases in the healthy group had an average age of 47.74 ± 21.27 (*P* = 0.549). The COVID-19 patients had a significantly higher body mass index (BMI) in comparison with healthy group (27.15 ± 5.09 and 22.17 ± 3.26, respectively; *P* = 0.000). Except for diabetes mellitus, there were no statistical differences in the frequency of underlying diseases among COVID-19 patients and healthy cases.

**Table 1:** Demographic characteristics of COVID-19 patients and healthy individuals.

Demographics characterizations	COVID-19 group				Control group N = 19	*P* value^#^
	Total N = 70	With GI symptoms N = 47	Without GI symptoms N = 23	*P* value*		
Gender, male	36 (51.4)	23 (48.9)	13 (56.5)	0.551	12 (63.2)	0.363
Age (years)	51.47 (± 17.28)	51.76 (± 17.80)	50.86 (± 16.47)	0.288	47.74 (± 21.27)	0.549
Age ≥ 65 years	19 (27.1)	14 (29.8)	5 (21.7)	0.123	4 (21.05)	0.243
BMI	27.15 (± 5.09)	26.96 (± 5.40)	27.52 (± 4.51)	0.684	22.17 (± 3.26)	**0.000**
**Underlying diseases**						
Hypertension	23 (32.9)	17 (36.2)	6 (26.1)	0.339	2 (10.53)	0.055
Diabetes mellitus	24 (34.3)	12 (25.5)	12 (52.2)	**0.027**	2 (10.53)	**0.043**
Cardiovascular disease	11 (15.7)	6 (12.8)	5 (21.7)	0.333	1 (5.26)	0.237
Dyslipidemia	5 (7.14)	3 (6.38)	2 (8.69)	0.724	0 (0)	0.243
Chronic kidney disease	8 (11.43)	6 (12.75)	2 (8.69)	0.615	1 (5.26)	0.429
Chronic liver disease	6 (8.57)	5 (10.63)	1 (4.34)	0.337	1 (5.26)	0.635
prior GI problems	9 (12.9)	9 (19.1)	0 (0)	**0.025**	5 (26.3)	0.153

Data are shown as frequency (percentage).

*COVID-19 patients with GI sign vs. no GI sign.

^#^COVID-19 patients vs. control. Bold text indicates *P* values < 0.05.

**Figure 1:**
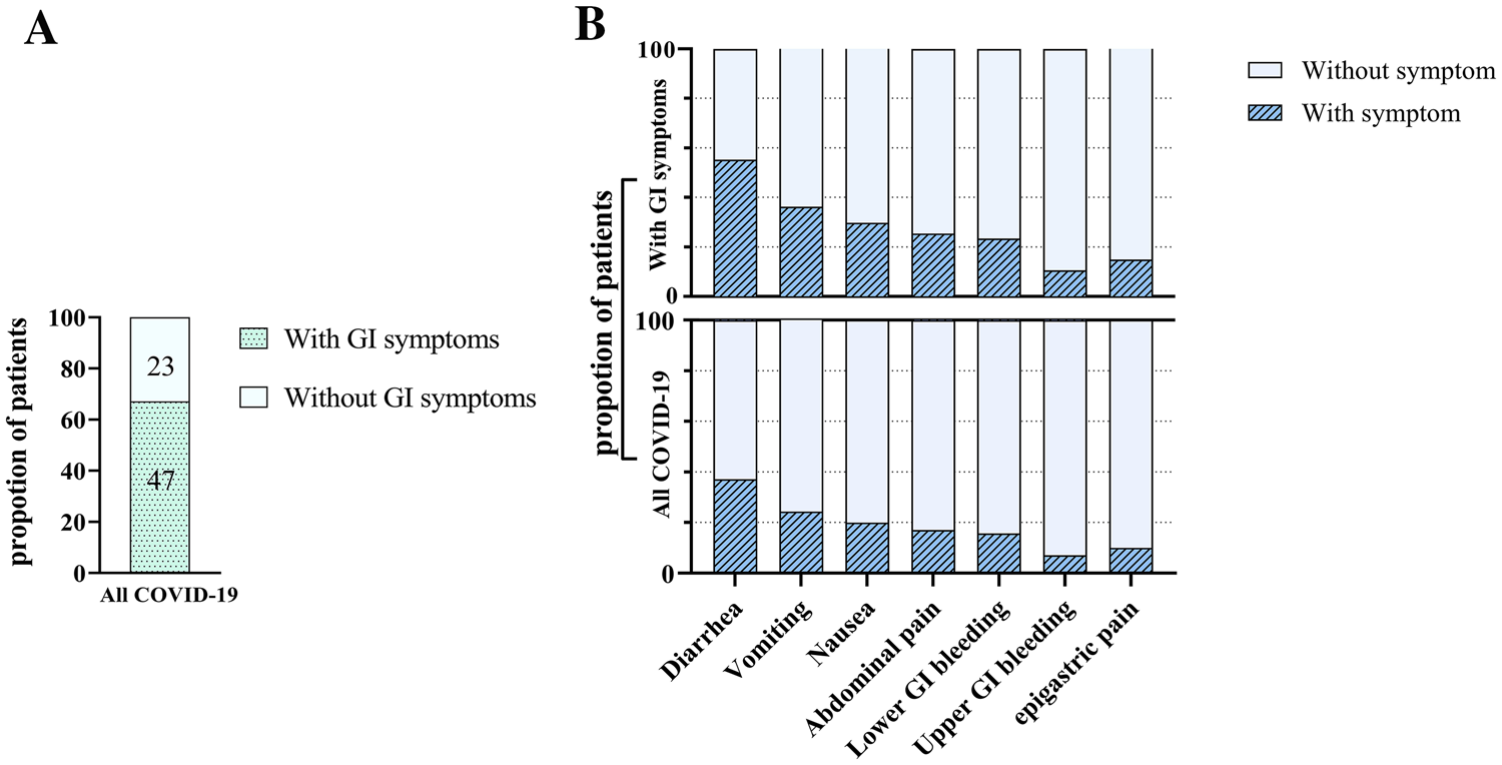
The proportion of patients based on GI symptoms. (**A**) The proportion of COVID-19 patients with or without GI symptoms. (**B**) Frequency of reported GI symptoms in both all patients and patients with GI symptoms.

Of 70 patients with COVID-19, 47 (67.14%) patients showed GI symptoms (Fig. 1A) with a mean age 51.76 ± 17.8 years, which were not significantly different compared to those who did not present any GI symptoms (50.86 ± 16.47 years), (*P* = 0.288). As shown in Table 1, the proportion of male patients as well as BMI did not differ significantly between patients with and without GI manifestations (*P* = 0.551 and *P* = 0.684, respectively). All features were statistically no different among the two distinct groups based on GI problems, except for diabetes mellitus and prior GI problems. The frequency of GI manifestations in Fig. 1B illustrates that diarrhea was the most reported GI symptom in 70 COVID-19 patients as well as in patients classified in the GI symptoms group (37.1% and 55.3%, respectively). Upper GI bleeding was the rarest GI symptom (7.1% and 10.6%, respectively).

COVID-19-associated clinical symptoms and hospitalization features of patients with and without GI symptoms are detailed in Table 2. Most patients were admitted to the hospital between the 7th and 9th day after the onset of symptoms. Coughing (91.3%) and fever (76.59%) were the most reported complaints in no GI and GI symptoms groups, respectively. Except for the higher frequency of cough (*P* = 0.010) in patients without GI symptoms, there were no statistical differences in COVID-19-associated clinical symptoms between patients with and without GI symptoms. Samples were collected during the acute phase of the disease. The median time from symptom onset to sampling was 9 days (9–13). Out of all the patients, 27.14% (n = 19) presented to the hospital with severe disease (see “Methods” for definitions); of these, five patients (7.17%) were admitted to the intensive care unit (ICU), four (5.71%) of whom died, and the other 66 patients were discharged after 7 (6–10) days. Distribution of disease severity was similar in patients with or without GI symptoms (*P* = 0.739); however, just GI group met an outcome of ICU admission (10.6%) and/or death (8.51%) compared to those without GI symptoms (*P* = 0.105 and *P* = 0.150, respectively). Other hospitalization characteristics between GI and no GI symptoms groups were also not statistically different.

**Table 2:** COVID-19 patients' features.

Clinical manifestations	COVID-19 group			
	Total	With GI symptoms	Without GI symptoms	*P* value
COVID-19-associated respiratory syndrome				
Cough	50 (71.4)	29 (61.7)	21 (91.30)	**0.010**
Dyspnea	47 (67.1)	31 (65.95)	16 (69.56)	0.763
Chest pain	11 (15.7)	7 (14.89)	4 (17.39)	0.787
Sore throat	9 (12.9)	7 (14.9)	2 (8.70)	0.467
Sputum	11 (15.7)	9 (19.14)	2 (8.70)	0.259
Tachypnea	6 (8.6)	5 (10.63)	1 (4.34)	0.377
Fever	54 (77.1)	36 (76.59)	18 (78.26)	0.876
Chills	43 (61.4)	29 (61.70)	14 (60.86)	0.946
Weakness	41 (58.6)	30 (63.82)	11 (47.82)	0.202
Myalgia	29 (41.4)	21 (44.7)	8 (34.8)	0.430
Headache	26 (37.1)	18 (38.29)	8 (34.78)	0.775
Dizziness	10 (14.3)	9 (19.14)	1 (4.34)	0.096
**Hospitalization characteristics**				
Onset of symptoms before admission to hospital, days	7 (4–9)	7 (4–10)	9 (6–12)	0.220
Serum and Fecal sampling after onset the symptoms, days	9 (6–13)	10 (6–13)	9 (6–12)	0.650
Duration of hospitalization,	7 (6–10)	7 (6–10)	7 (6–12)	0.804
Severe illness	19 (27.14)	14 (29.8)	5 (21.7)	0.739
ICU admission	5 (7.17)	5 (10.6)	0 (0)	0.105
Death	4 (5.71)	4 (8.51)	0 (0)	0.150

Data are presented frequency (percentage) or median and 25th and 75th percentiles (P25–P75) for variables with non-normal distribution. Bold font indicates statistical significance at the *P* value < 0.05 level.

**Figure 2:**
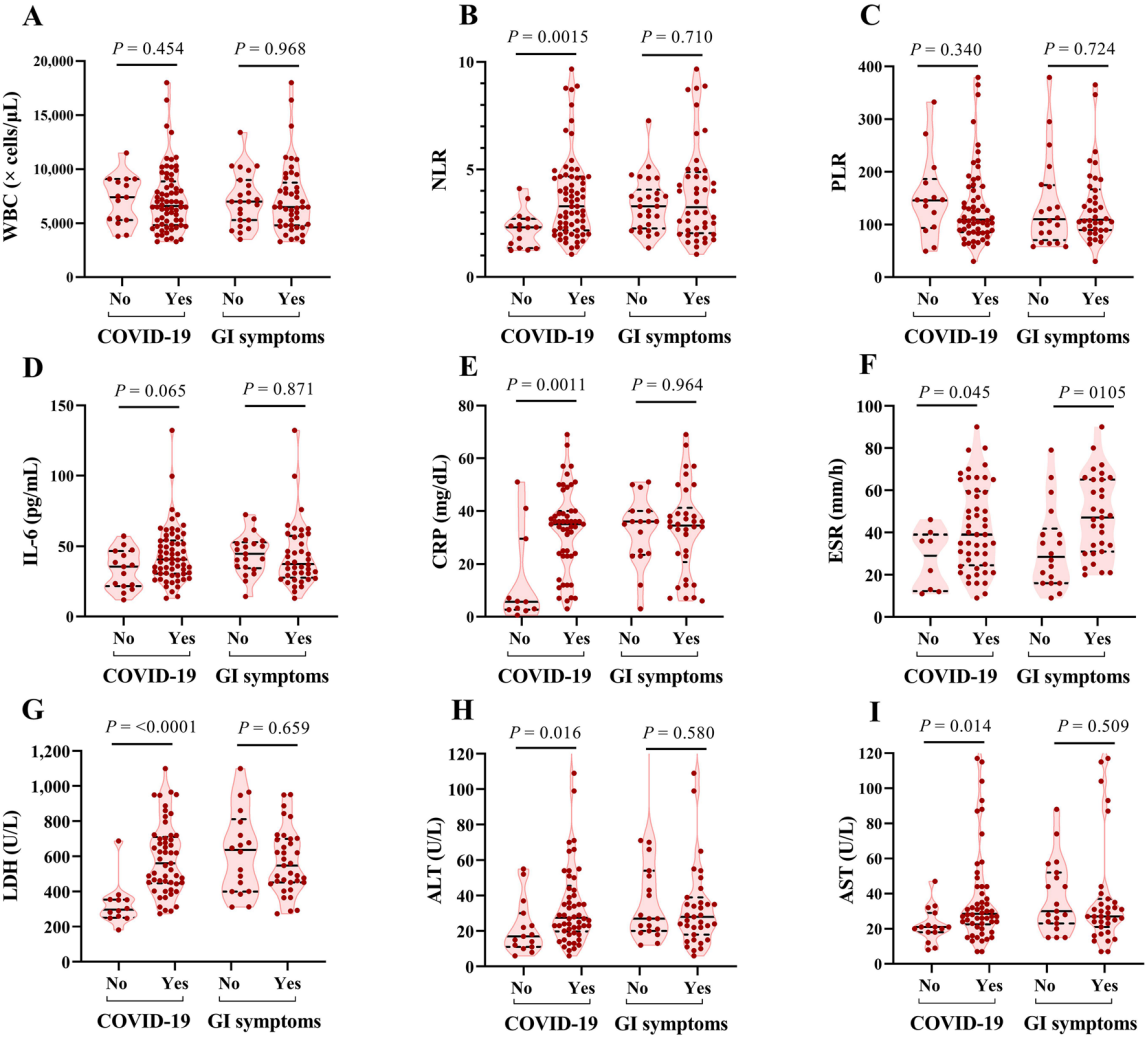
Laboratory findings of COVID-19 and healthy individuals. Hematology and biochemistry markers in COVID-19 patients compared with control. Comparisons between patients with and without GI symptoms are also shown. The median and the interquartile ranges are represented on violin plots. Each point indicates a value of a patient. The *P* values calculated using the Mann–Whitney test and Welch t-test with significance defined as *P* < 0.05.

According to laboratory findings illustrated in Fig. 2, some parameters including neutrophil–lymphocyte ratio (NLR), CRP, ESR, LDH, ALT and AST were significantly higher in COVID-19 patients compared with the control group. However, there were no significant differences between COVID-19 patients with and without GI symptoms, except that those with GI symptoms tended to have higher ESR values. Other markers analyzed (WBC, PLR, IL-6) were not significantly different between controls and COVID-19 patients nor associated with GI symptoms. No other notable differences in hematological parameters (Hb, lymphocytes, neutrophils, and platelets) and biochemical values (CPK, Alb, Urea, Cr, and PT) were seen in those with GI symptoms compared to those without (Data not shown).

### FC and SC were increased in COVID-19 patients but were unrelated to GI symptoms

**Figure 3:**
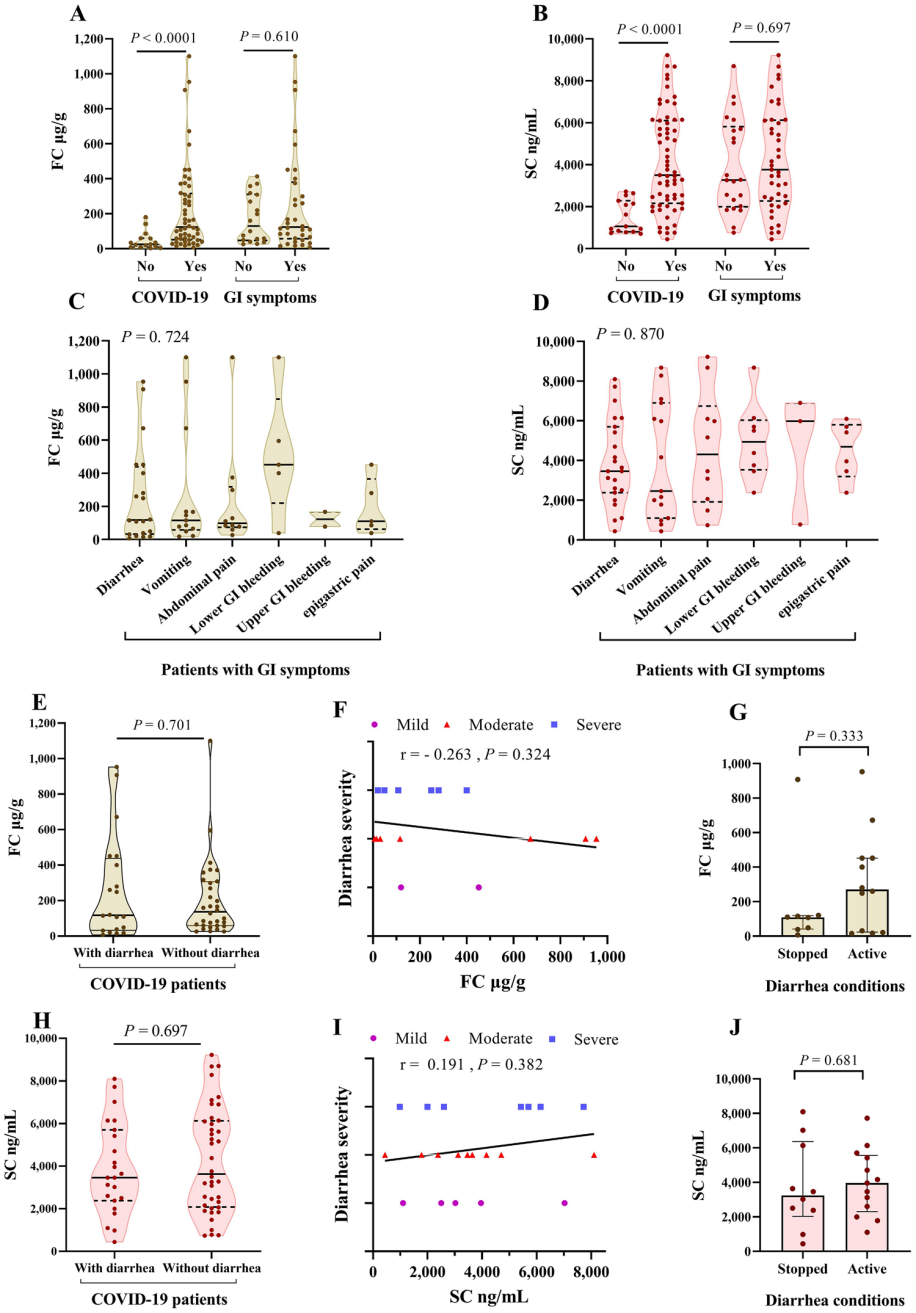
Concentrations of SC and FC in COVID-19 and healthy donors. Comparison of FC and SC concentrations based on COVID-19 infection as well as having GI symptoms (**A**,**B**). SC and FC levels based on the types of GI problems in patients with GI symptoms (**C**,**D**). SC and FC concentrations in COVID-19 patients based on the presence of diarrhea (**E**,**H**). The correlation of FC and SC with diarrhea severity (**F**,**I**). Comparison of FC and SC levels between two diarrhea conditions (**G**,**J**). Plots show median ± interquartile range, and *P* values are shown in the figures.

The association of FC with viral or bacterial gastroenteritis^17^ and fecal SARS-CoV-2 RNA detection by previous reports^18^ led us to investigate if COVID-19 infection causes GI inflammation. We measured concentrations of FC and SC in the stool and serum samples from COVID-19 patients as well as control group. Levels of FC or SC showed a significant increase in COVID-19 patients compared with uninfected controls (Fig. 3A,B). Interestingly no differences were found in FC and SC levels between those patients reporting GI symptoms compared to those without GI symptoms (124.25 vs. 130 µg/g, and 3760 vs. 3270 ng/mL, respectively). Among 52.9% COVID-19 patients with elevated FC, 73.5% had GI symptoms, and 66.7% no GI symptoms. The same results were found for SC in terms of 74.3% in the infected group, so 85.4% with and 77.3% without GI symptoms.

To further investigate the FC and SC concentrations in relation to GI symptoms, we compared FC or SC levels depending on the types of GI symptoms reported in patients who presented GI complaints. Kruskal–Wallis test showed no significant difference in FC or SC between diarrhea, vomiting, and other shown in Fig. 3C,D. In the next analysis, patients were classified into two categories based on the presence or absence of diarrhea. This comparison also exhibited no difference between the two categories (Mann–Whitney test) (Fig. 3E,H). Spearman correlation then revealed non-significant relationship between the severity of diarrhea (mild, moderate, severe) with FC (r = − 0.263) or SC (r = 0.191) variables (Fig. 3F,I).

Considering the hypothesis that the increase in FC may be related to the concurrence of diarrhea during sampling, we distinguished the values of FC and SC in two conditions; 14 patients were classified as active diarrhea and the remaining 11 patients had stopped diarrhea during sampling. Of 25 diarrhetic subjects, we collected stool samples from 20 and serum samples from 23, and only these patients are considered in these analyses. Figure 3G,J show that FC levels were higher in patients with active diarrhea but were not significantly different from those who reported diarrhea before stool sampling (270 vs. 109 µg/g). The values of SC in both conditions did not show a difference (3240 vs. 3960 ng/mL). We also analyzed this comparison based on whether diarrhea was the first symptom in patients or not, and again no significant difference was observed in either FC or SC (Data not shown).

### FC was correlated with SC in COVID-19 while not related to other laboratory parameters

**Figure 4:**
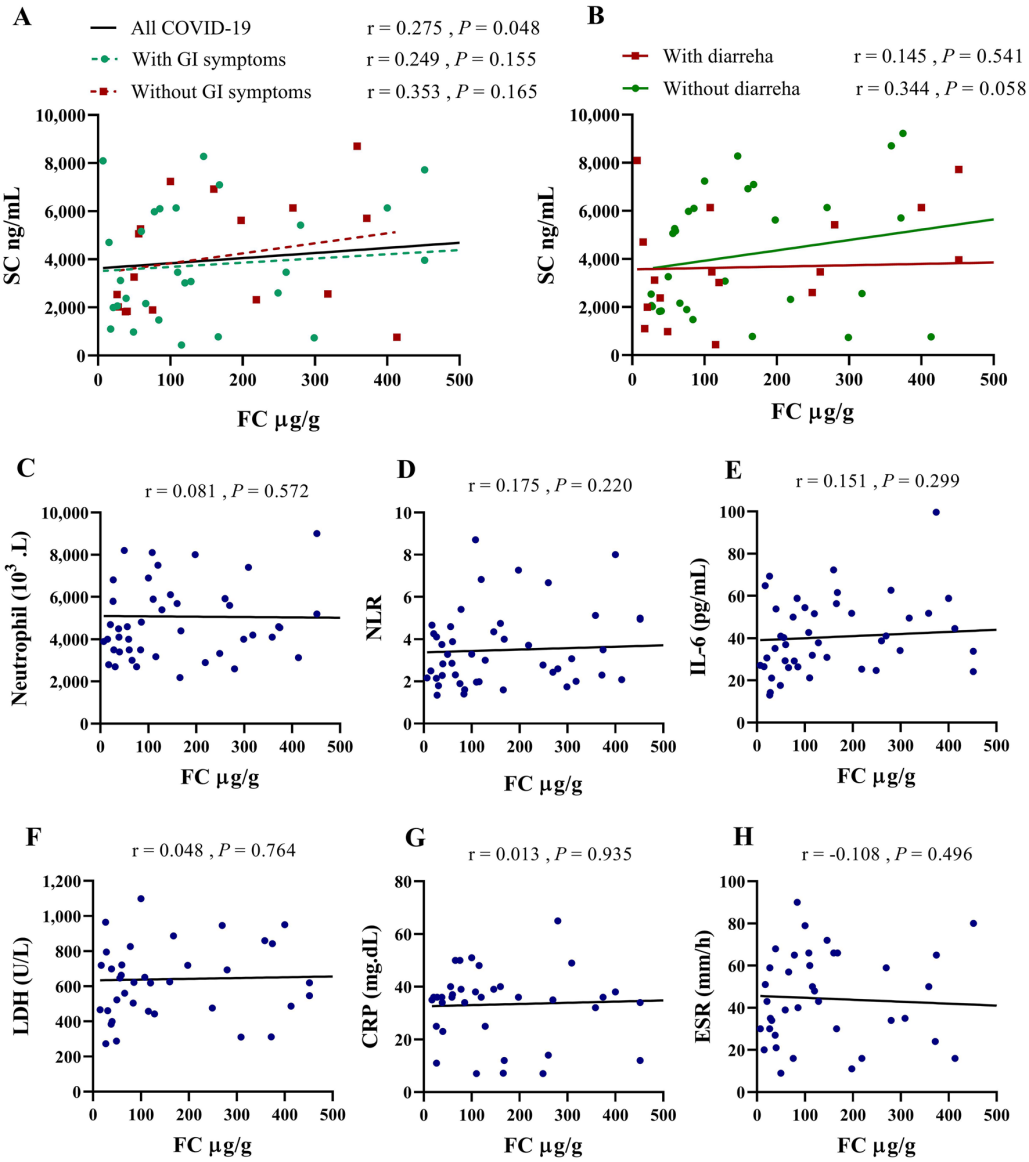
Correlation of FC with clinical laboratory studies. Data were examined by Pearson correlation and Spearman correlation for normal and non-normal data. Correlation coefficients and *P* values are shown in the figures.

Spearman correlation showed a significant positive relationship between FC and SC variables in COVID-19 patients (*P* = 0.048); however, the same correlation was not observed with the presence of GI symptoms. As shown in Fig. 4A, there was no significant relationship between FC and SC in COVID-19 groups with or without GI symptoms. It was also found that FC and SC were not associated with COVID-19 patients presenting with and without diarrhea (Fig. 4B).

We next determined whether FC is related to commonly available laboratory parameters. In particular, potential correlations with the absolute neutrophil count, absolute lymphocyte count, NLR, IL-6, CRP, and ESR were assessed. As seen in Fig. 4C–H, there was no significant relationship between FC level and other parameters in the whole study population. Similar results were observed in the same analysis in COVID-19 patients with and without GI symptoms (Data not shown).

### Elevated FC is associated with worsened clinical status

**Figure 5:**
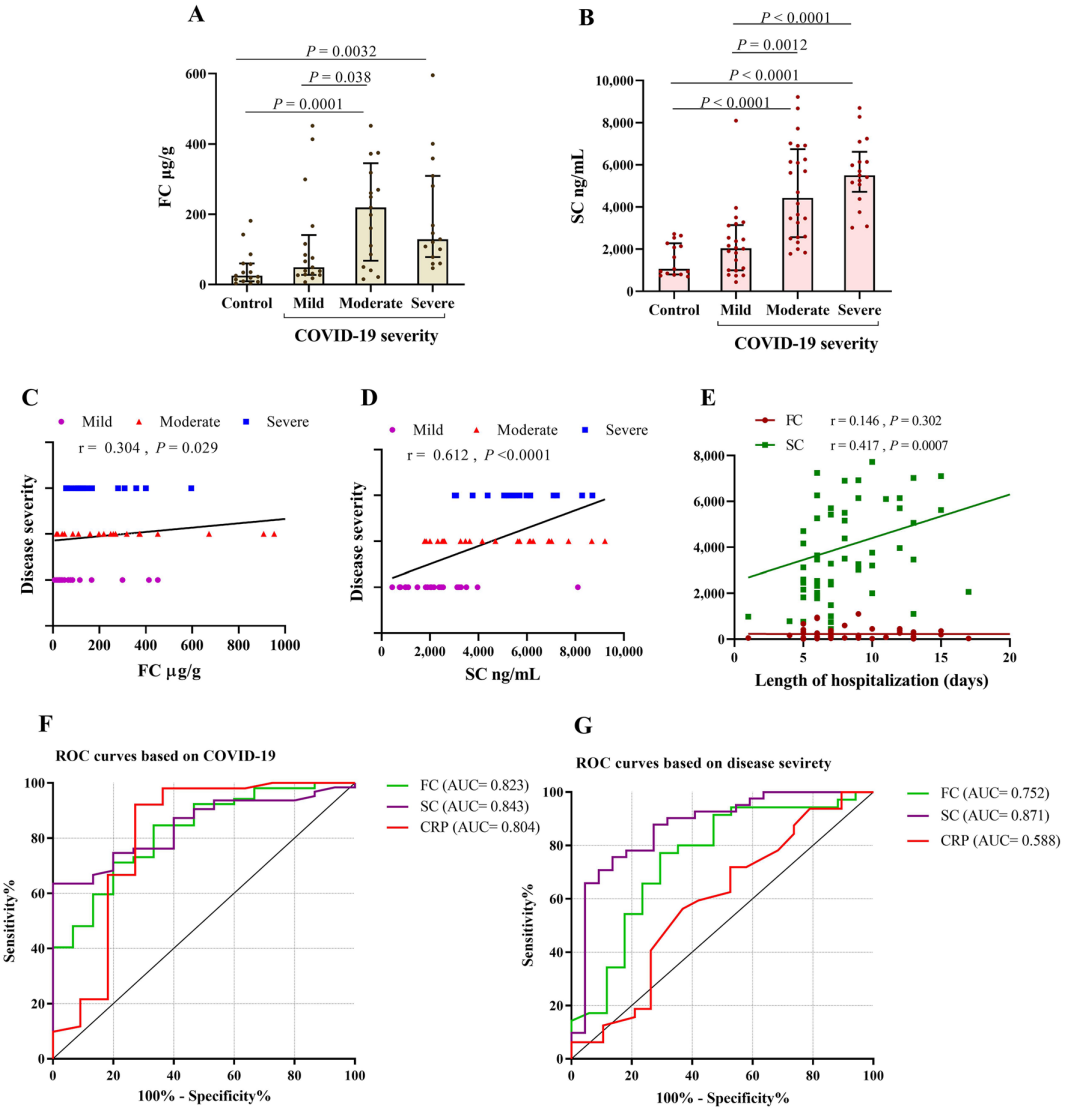
FC and SC relation to COVID-19 severity. Comparison of SC and FC levels according to three classes of disease severity (**A**,**B**). The correlation of FC or SC with disease severity (**C**,**D**). The correlation of FC or SC with the duration of hospitalization (**E**). Receiver operating characteristic (ROC) curve evaluation of the performance of FC, SC and CRP risk score in diagnosis COVID-19 patients or distinguish severe cases (**F**,**G**). AUC: under the ROC curve. *P* values are shown in the figures.

Analysis of FC and SC in COVID-19 patients stratified by disease severity showed that FC and SC levels were highest in moderate (254 µg/g and 4430 ng/mL, respectively) and severe (128 µg/g and 5500 ng/mL, respectively) form of the disease. As illustrated in Fig. 5A,B, FC and SC concentrations were significantly different among control and severity stratifications (Kruskal–Wallis test). This analysis suggested that raised FC and SC among these patients may be the feature of severe disease. To confirm this possibility, we next assessed the correlation between FC or SC and disease severity. A striking positive association was found for SC (*P* < 0.0001), but a less robust association was appreciated for FC (*P* < 0.029) (Fig. 5C,D).

The length of hospitalization and mortality are considered as the criteria for illness severity^19^. No correlation between FC and length of hospital stay was observed, and conversely for SC (Fig. 5E). Analysis of FC or SC revealed no relationship with discharge or death described as disease outcome (*P* = 0.415 and *P* = 0.076, respectively). Furthermore, considering the presence of GI symptoms, there was no significant relationship between FC or SC and diarrhea severity (*P* = 0.825 and *P* = 0.382, respectively), nor in the presence of diarrhea (*P* = 0.985 and *P* = 0.698, respectively) (Data not shown).

ROC curve was performed to further evaluate the clinical utility of studied markers and determine the optimum cut-off point for differentiation between COVID-19 patients and control individuals. We found that the biggest area under the ROC curve (AUC) belonged to SC (0.842, 95% CI 0.753–0.932, *P* = 0.000) and FC (0.823, 95% CI 0.709–0.936, *P* = 0.000) as compared with the other markers which had slightly lower AUCs; CRP (0.803, 95% CI 0.613–0.994, *P* = 0.001) (NLR and ESR with lower AUCs were not shown) (Fig. 5F). Patients with low or high levels were characterized according to the concentrations of FC at a cut-off of > 58.38 µg/g with a sensitivity of 73.1% and a specificity of 73.3%. The cut-off points were calculated for SC at 1950 ng/mL (sensitivity 81.0% and specificity 60%), and CRP at 7.150 mg/dL (sensitivity 92.2% and specificity 72.7%).

ROC curve was also used to differentiate between severe COVID-19 and non-severe COVID-19 patients and examine the prognostic value of the studied markers. The results showed that the AUC was 0.752 (95% CI 0.600–0.900, *P* = 0.004) for FC and 0.871 (95% CI 0.773–0.968, *P* < 0.0001) for SC which were higher than CRP 0.588 (95% CI 0.419–0.757, *P* = 0.297). Severe patients were presented by a cut-off > 84.78 µg/g for FC, > 3230 ng/mL for SC, and > 34.50 mg/dL for CRP.

A binary logistic regression test was used to predict the severity and outcome of the COVID-19 disease. The results showed that FC and SC levels could be used as predictors of COVID-19 disease severity (OR 1.005, 95% CI 1.0003–1.0098, *P* = 0.0368 and OR 1.001, 95% CI 1.0004–1.0013, *P* = 0.0001, respectively). The results also showed that FC could not predict the outcome of COVID-19 disease (OR 1.000, 95% CI 0.996–1.004, *P* = 0.879).

## Discussion

This prospective study is presenting the first evidence of simultaneous FC and SC level in COVID-19 patients. FC and SC were increased robustly in COVID-19 patients compared with the control group, with higher levels in severe subjects. Elevated levels of FC may indicate GI tract inflammation in COVID-19 cases, but no significant difference was observed in the presence of GI symptoms. Also, the higher NLR, CRP, ESR, LDH as well as SC concentrations in COVID-19 patients reflect the systemic immune-inflammation as a part of SARS-CoV-2 infection. Despite the coincidence of diarrhea or other GI symptoms and FC raising, these responses could not prove GI cells inflammation, given that none of above-mentioned inflammatory markers were notably elevated in patients with GI symptoms.

Across our study, the main GI symptom presented in 26/70 (37.1%) of COVID-19 patients was diarrhea; consistent with the previous studies in which diarrhea was a frequent presenting symptom in patients infected with SARS-CoV-2^20^. Viral RNA detection in gastrointestinal epithelium and feces from COVID-19 patients who presented with diarrhea supports gut infectivity and incidence of diarrhea^8,21,22^. In this regard, Effenberger et al. reported that SARS-CoV-2 infection triggered the gut inflammatory response, as evidenced by diarrhea and elevated FC. However, a very high FC characterized during acute diarrhea was not accompanied with viral RNA detection in feces^23^. In contrast, our result, similar to Britton et al.^8^, showed that FC had no relation to diarrhea, so that FC was not higher in COVID-19 patients with diarrhea. Also, FC didn’t associate even with diarrhea condition and its elevation was not significant in those who had active diarrhea compared to those with stopped form. Two possibilities were proposed here: first, diarrhea was not caused by inflammation which is secondary to enterocyte invasion and destruction. Second, FC elevation was not due to GI inflammation. There is little histological evidence of apparent intestinal inflammation to support GI inflammation^24^. According to our data, the lack of FC correlation with diarrhea may be attributed to diarrhea resulting from a non-inflammatory mechanism, which is mostly caused by viral etiology^25^. Moreover, no fecal leukocytes were found among our diarrheal patients, which was consistent with viral diarrhea characteristics and non-inflammatory nature^25^. Thus, it can be noted that diarrhea does not necessarily coincide with FC raising.

We found that FC positively correlated with SC, however, no correlation with any of GI symptoms. This data may suggest that SARS-CoV-2 could also cause GI manifestations without direct invasion to GI cells supported by negative stool qPCR in nearly half of COVID-19 patients who presented GI symptoms^8,22,26,27^. Moreover, FC elevation in COVID-19 may not represent a localized response within the intestinal infection by SARS-CoV-2 but likely reflect a systemic immune-response caused by immune cells recruitment to the GI tract, contributing to producing and releasing calprotectin through the gut^2,14^. On the other hand, circulating inflammatory cytokines can induce cellular infiltration of the intestinal wall^28^, which can cause FC secretion itself. There are still gaps in understanding the mechanisms through which SARS-CoV-2 causes diarrhea and whether COVID-19-induced inflammatory response makes diarrhea.

GI involvement has been proposed to alleviate COVID-19 severity and mortality with an accompanying decrease in serum inflammatory cytokines levels^29^. Our observations inversely revealed that severe COVID-19 requiring ICU admission all had GI symptoms, and 80% of them eventually died. On the other hand, a mild/severe disease course was not found related to GI symptoms, however, Cheung et al. reported that GI symptoms were higher in patients with severe disease than in patients with mild disease^26^. As a consequence, there is disagreement among various reports about the relationship between GI symptoms and disease severity^24^.

Furthermore, we did find high levels of SC in patients who were ongoing clinical worsening, consistent with previous studies that already indicated the predictive value of SC to distinguish severe COVID-19^8,30–33^. Though our study similar to some others^8,15,23^ found an increase in FC, however, this is one of the first to demonstrate that high FC levels correlate with COVID-19 disease. It seems FC has a good discrimination capacity, as assessed by the analysis of the AUC of ROC curve for COVID-19 diagnosis and severity prognosis, similar to SC but numerically lower than it. Indeed, FC and SC both are valuable prognostic markers, as disease progression is marked by FC and SC increases. In our theory, if FC or SC elevation had been associated with GI symptoms, SC could be suggested as a strong biomarker for assessing GI inflammation in COVID-19 infection by the following reasons: SC had the highest predictive value for COVID-19, FC and SC had the same correlation with the extent and prognosis of IBD^13^, in accordance with COVID-19 disease found here. Anyway, we hope to have contributed to the development of future studies.

This report has some limitations. The sample size was relatively small, though the patients were from two centers. However, multicenter research might be a disadvantage since the heterogeneity might be amongst different laboratory centers. Albeit, biomarkers of interest such as FC, SC, IL-6 were examined through a specially experienced team. In addition, FC and SC were measured only once at the acute phase of the disease, however, another sampling after remission might present exclusive data.

## Conclusions

To our knowledge, this is the first study regarding the relationship between FC and SC in COVID-19. Our data suggest that FC and SC might have the potency to assess the prognosis in COVID-19 patients, but increased FC and SC did not feature GI symptoms or even diarrhea in COVID-19. Whether the expression of SARS-COV-2 receptors in the GI tract is related to calprotectin levels despite diarrhea and/or of GI symptoms would be an attitude for the next efforts. Finally, these results would reinforce therapeutic approaches targeting calprotectin to attenuate severe COVID-19.

## Methods

### Patients

Between September and November 2020, stool and serum samples were collected from COVID-19 patients admitted to the two main centers of COVID-19 in Mazandaran Province, Razi and Imam Hospitals. SARS-CoV-2 infection was confirmed by nasopharyngeal swab using qPCR and clinical confirmation based on chest CT scan findings. COVID-19-confirmed patients under 18 years of age, patients with diarrhea that had simultaneous viral, bacterial, parasitic, and protozoan GI infection, and those who did not give written informed consent were excluded from the study. Also, no samples were taken from COVID-19 patients with inflammatory bowel diseases and autoimmune diseases. Finally, included patients were categorized based on qualifying GI symptoms including diarrhea, nausea, vomiting, abdominal pain, upper or lower GI bleeding, and epigastric pain into two groups: with GI symptoms (*n* = 47) and without GI symptoms (*n* = 23). 19 healthy individuals, age-matched, were selected as the control group whose had negative qPCR for COVID-19. All participant were clearly informed prior to their inclusion in the study.

GI symptoms must have persisted for at least one day prior to enrollment. Loose stool more than 3 times per day was considered diarrhea^34^. Date of diarrhea was also recorded as the following conditions; Stopped diarrhea (patients who had diarrhea before sampling) and Active diarrhea **(**patients with diarrhea during sampling).

Disease severity was defined at the time of admission as mild (early infection, non-pneumonia, and/or minor lung involvement), moderate (pneumonia with lungs involvement < 50%), and severe (distressed/dyspneic, lungs infiltration > 50%) according to the WHO-China Report for COVID-19^35^. Clinical outcome was pointed as death or discharge from the hospital.

The Ethics Committee of Mazandaran University of Medical Sciences approved this non-interventional study (IR.MAZUMS.IMAMHOSPITAL.REC.1399.035). Written consent was obtained from all participants in this study. All methods were carried out in accordance with relevant guidelines and regulations.

### Samples collection

Blood and stool samples were collected from each individual at admission and/or throughout hospitalization when they were in the acute phase of the disease. Separated serum was kept at – 70 °C and then used to measure SC, and other biochemical factors. The stool samples were collected into clean plastic containers, registered and stored at – 70 °C, then used for FC measurement.

### Laboratory measurements

FC and SC were measured using the CALPROLAB™ ELISA (HRP) method (CALPRO AS, Lysaker, Norway) according to the manufacturer's instruction. The concentration of calprotectin was expressed as µg/g for FC and ng/mL for SC. All other parameters, including complete blood count (CBC), hemoglobin (Hb), IL-6, C-reactive protein (CRP), erythrocyte sedimentation rate (ESR), lactate dehydrogenase (LDH), aspartate aminotransferase (AST), and alanine aminotransferase (ALT), were determined in the hospitals laboratories by routine laboratory analysis.

### Statistical analysis

Statistical analyses were performed using SPSS (V.25, Chicago, IL, USA) and GraphPad Prism (V.9.1.0.221, GraphPad, Inc.). Data with normal distribution were shown as mean ± SD, and non-normal data were illustrated as median and 25th and 75th percentiles (P25–P75). Welch t-test was used to perform mean comparisons for normally distributed data. Mann–Whitney and Kruskal–Wallis tests were used for the analysis of non-normal data. Correlations between variables were measured using Pearson correlation and Spearman rank correlation tests for normal and non-normal data, respectively. Potential clinical utility of calprotectin based on COVID-19 infection was evaluated using Receiver Operating Curve (ROC) analysis. The predictive value of calprotectin for disease severity was calculated using binary logistic regression analysis. *P* ˂ 0.05 was considered statistically significant.

## References

[1] Zhu N (2020). A novel coronavirus from patients with pneumonia in China, 2019. N. Engl. J. Med..

[2] Gu J, Han B, Wang J (2020). COVID-19: Gastrointestinal manifestations and potential fecal–oral transmission. Gastroenterology.

[3] Jin Y-H (2020). A rapid advice guideline for the diagnosis and treatment of 2019 novel coronavirus (2019-nCoV) infected pneumonia (standard version). Mil. Med. Res..

[4] Wong Sunny H, Lui Rashid Ns, Sung Joseph Jy (2020). Covid-19 and the digestive system. JGH Open.

[5] Huang R (2020). Clinical findings of patients with coronavirus disease 2019 in Jiangsu province, China: A retrospective, multi-center study. PLoS Negl. Trop. Dis..

[6] Kouhsari E (2020). Clinical, epidemiological, laboratory, and radiological characteristics of novel Coronavirus (2019-nCoV) in retrospective studies: A systemic review and meta-analysis. Indian J. Med. Microbiol..

[7] Redd WD (2020). Prevalence and characteristics of gastrointestinal symptoms in patients with SARS-CoV-2 infection in the United States: A multicenter cohort study. Gastroenterology.

[8] Britton GJ (2020). SARS-CoV-2-specific IgA and limited inflammatory cytokines are present in the stool of select patients with acute COVID-19. medRxiv.

[9] Liao M (2020). Single-cell landscape of bronchoalveolar immune cells in patients with COVID-19. Nat. Med..

[10] Silvin A (2020). Elevated calprotectin and abnormal myeloid cell subsets discriminate severe from mild COVID-19. Cell.

[11] Udeh R, Advani S, de Guadiana Romualdo LG, Dolja-Gore X (2021). Calprotectin, an emerging biomarker of interest in COVID-19: A systematic review and meta-analysis. J. Clin. Med..

[12] Kaya T, Yaylacı S, Nalbant A (2021). Serum calprotectin as a novel biomarker for severity of COVID-19 disease. Ir. J. Med. Sci.

[13] Kopi TA, Shahrokh S, Mirzaei S, Aghdaei HA, Kadijani AA (2019). The role of serum calprotectin as a novel biomarker in inflammatory bowel diseases: A review study. Gastroenterol. Hepatol. Bed.

[14] Ayling RM, Kok K (2018). Fecal calprotectin. Adv. Clin. Chem..

[15] Ojetti V (2020). COVID-19 and intestinal inflammation: Role of fecal calprotectin. Dig. Liver Dis..

[16] Jena A, Kumar-M P, Singh AK, Sharma V (2020). Fecal calprotectin levels in COVID-19: Lessons from a systematic review on its use in inflammatory bowel disease during the pandemic. Dig. Liver Dis..

[17] Havelka A, Sejersen K, Venge P, Pauksens K, Larsson A (2020). Calprotectin, a new biomarker for diagnosis of acute respiratory infections. Sci. Rep..

[18] Wong MC (2020). Detection of SARS-CoV-2 RNA in fecal specimens of patients with confirmed COVID-19: A meta-analysis. J. Infect..

[19] Huang J (2021). Risk stratification scores for hospitalization duration and disease progression in moderate and severe patients with COVID-19. BMC Pulm. Med..

[20] D’Amico F, Baumgart DC, Danese S, Peyrin-Biroulet L (2020). Diarrhea during COVID-19 infection: Pathogenesis, epidemiology, prevention and management. Clin. Gastroenterol. Hepatol..

[21] Zhang Y (2021). Prevalence and persistent shedding of fecal SARS-CoV-2 RNA in patients with COVID-19 infection: A systematic review and meta-analysis. Clin. Transl. Gastroenterol..

[22] Xiao F (2020). Evidence for gastrointestinal infection of SARS-CoV-2. Gastroenterology.

[23] Effenberger M (2020). Faecal calprotectin indicates intestinal inflammation in COVID-19. Gut.

[24] Guo M, Tao W, Flavell RA, Zhu S (2021). Potential intestinal infection and faecal–oral transmission of SARS-CoV-2. Nat. Rev. Gastroenterol. Hepatol..

[25] Barr W, Smith A (2014). Acute diarrhea in adults. Am. Fam. Physician.

[26] Cheung KS (2020). Gastrointestinal manifestations of SARS-CoV-2 infection and virus load in fecal samples from a Hong Kong cohort: Systematic review and meta-analysis. Gastroenterology.

[27] Lin L (2020). Gastrointestinal symptoms of 95 cases with SARS-CoV-2 infection. Gut.

[28] Reisinger EC, Fritzsche C, Krause R, Krejs GJ (2005). Diarrhea caused by primarily non-gastrointestinal infections. Nat. Clin. Pract. Gastroenterol. Hepatol..

[29] Livanos AE (2020). Gastrointestinal involvement attenuates COVID-19 severity and mortality. MedRxiv.

[30] Bauer W (2020). Outcome prediction by serum calprotectin in patients with COVID-19 in the emergency department. J. Infect..

[31] Chen L (2020). Elevated serum levels of S100A8/A9 and HMGB1 at hospital admission are correlated with inferior clinical outcomes in COVID-19 patients. Cell. Mol. Immunol..

[32] de GuadianaRomualdo LG (2020). Circulating levels of GDF-15 and calprotectin for prediction of in-hospital mortality in COVID-19 patients: A case series. J. Infect..

[33] Shi H (2021). Neutrophil calprotectin identifies severe pulmonary disease in COVID-19. J. Leukoc. Biol..

[34] Chiejina, M. & Samant, H. Viral Diarrhea. *StatPearls [Internet]* (2020). 29262044

[35] Gomes C (2021). Report of the WHO-China joint mission on coronavirus disease 2019 (COVID-19). Braz. J. Implantol. Health Sci..

